# Blink rate and facial orientation reveal distinctive patterns of attentional engagement in autistic toddlers: a digital phenotyping approach

**DOI:** 10.1038/s41598-023-34293-7

**Published:** 2023-05-03

**Authors:** Pradeep Raj Krishnappa Babu, Vikram Aikat, J. Matias Di Martino, Zhuoqing Chang, Sam Perochon, Steven Espinosa, Rachel Aiello, Kimberly L. H. Carpenter, Scott Compton, Naomi Davis, Brian Eichner, Jacqueline Flowers, Lauren Franz, Geraldine Dawson, Guillermo Sapiro

**Affiliations:** 1 Department of Electrical and Computer Engineering, Duke University, Durham, NC USA; 2 Department of Computer Science, Duke University, Durham, NC USA; 3 Ecole Normale Supérieure Paris-Saclay, Gif-Sur-Yvette, France; 4 Office of Information Technology, Duke University, Durham, NC USA; 5 Department of Psychiatry and Behavioral Sciences, Duke University, Durham, NC USA; 6 Duke Center for Autism and Brain Development, Duke University, Durham, NC USA; 7 Department of Pediatrics, Duke University, Durham, NC USA; 8 Duke Global Health Institute, Duke University, Durham, NC USA; 9 Departments of Biomedical Engineering, Mathematics, and Computer Science, Duke University, Durham, NC USA

**Keywords:** Biomarkers, Health care, Engineering, Computer science

## Abstract

Differences in social attention are well-documented in autistic individuals, representing one of the earliest signs of autism. Spontaneous blink rate has been used to index attentional engagement, with lower blink rates reflecting increased engagement. We evaluated novel methods using computer vision analysis (CVA) for automatically quantifying patterns of attentional engagement in young autistic children, based on facial orientation and blink rate, which were captured via mobile devices. Participants were 474 children (17–36 months old), 43 of whom were diagnosed with autism. Movies containing social or nonsocial content were presented via an iPad app, and simultaneously, the device’s camera recorded the children’s behavior while they watched the movies. CVA was used to extract the duration of time the child oriented towards the screen and their blink rate as indices of attentional engagement. Overall, autistic children spent less time facing the screen and had a higher mean blink rate compared to neurotypical children. Neurotypical children faced the screen more often and blinked at a lower rate during the social movies compared to the nonsocial movies. In contrast, autistic children faced the screen less often during social movies than during nonsocial movies and showed no differential blink rate to social versus nonsocial movies.

## Introduction

A large body of literature has utilized eye tracking to document differences in gaze patterns to social versus nonsocial stimuli in autistic individuals across the lifespan^1–3^. While the majority of studies of attention in autism have focused on gaze patterns, spontaneous eye blink rate has also been used to assess attention^4^. Studies have demonstrated task-related modulation of blink rate, with rate of blinking inversely related to level of encoding of information in working memory and attentional engagement ^5–7^. The evolutionary basis of varying blink rate stems from the idea that real-time assessments of the salience and value of information unconsciously change blink rate to increase or decrease the amount of visual information that is processed^8^. Evidence suggests a connection between spontaneous blink rate and striatal dopamine activity, with decreased blink rate found in persons with Parkinson’s disease, attention-deficit/hyperactivity disorder (ADHD), and fragile X syndrome^9–11^. Hornung et al.^12^ found that, compared to neurotypical children, blink rate and theta spectral EEG power, another measure of attentional engagement, were both reduced in autistic children. Another study using eye tracking found that neurotypical children exhibited lower blinking when watching scenes with high affective content, whereas autistic children blinked less frequently when looking at physical objects^13^. These results are consistent with findings that autism is associated with reduced social attention^1^, which is evident as early as 2–6 months of age^14,15^.

Traditionally, eye tracking has been used to measure gaze and blink rate patterns. We explored whether it was possible to detect meaningful patterns of attention via blink rate in toddlers using computer vision analysis (CVA) based on data collected via an application (app) on a smart tablet without the use of additional equipment. In a previous study, we demonstrated that it was possible to reliably measure atypical patterns of gaze, characterized by reduced attention to social stimuli, via CVA in young autistic toddlers compared to their neurotypical peers^16^.

The current analysis extends previous work by studying blink rate as an additional method for capturing patterns of attentional engagement in toddlers while they watched a series of strategically-designed social and nonsocial movies on a smart tablet. Along with blink rate, we also estimated the duration of the child orienting towards the tablet’s screen, denoted as total time facing forward (TFF). We predicted that neurotypical toddlers would reduce their blinking and thus exhibit lower blink rate when viewing movies with high social content, as compared to those without social content. In contrast, we predicted that autistic toddlers would either fail to exhibit a differential blink rate to movies with social versus nonsocial content or show lower blink rates when viewing movies with nonsocial content, suggesting higher attentional engagement when viewing nonsocial stimuli.

## Results

### Effects of group and stimulus type on facing forward and blink rate variables

**Figure 1:**
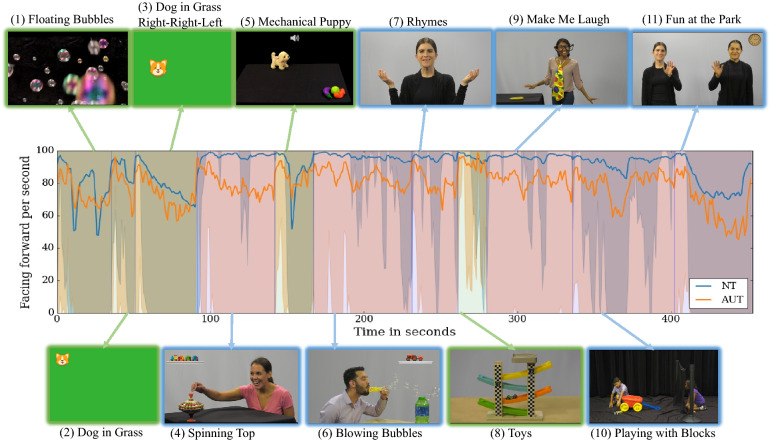
Representation of the ‘facing forward’ variable for the participants along with snapshots of the presented movies. The blue and green semi-transparent areas in the plot represent the time segments of the respective social and nonsocial movies. The line plot in the middle shows the mean of ‘facing forward’ with the 5th and 95th percentile among the neurotypical (NT) and autistic (AUT) groups for a one second window.

To estimate the main effects of group and stimulus type (social versus nonsocial movies) and their interaction effects for total time facing forward (TFF) and blink rate, a 2X2 mixed ANOVA was conducted. This analysis was based on the movies that had primarily social or nonsocial content (refer to the “Methods and materials” section along with Fig. 1 for details of the movies presented in the app). Mean TFF and mean blink rate were estimated for both the social and nonsocial movies. “Blowing Bubbles” and “Spinning Top” were excluded during this analysis since they contain both social and nonsocial content (see Fig. 1). Figure 1 depicts the mean with 5th and 95th percentile of the time-series associated with the ‘facing forward’ variable per one second window (see “Methods and materials” for details on the computation of ‘facing forward’). The distributions associated with the neurotypical/autistic groups are shown in blue/orange. Moments of presentation of social and nonsocial movies are highlighted with blue and green (respectively) semitransparent boxes.

A main effect of group was found for mean TFF (*F* (1, 440) = 40.76, *P* < 0.0001, η_p_^2^ = 0.086) and mean blink rate (*F* (1, 440) = 17.63, *P* < 0.0001, η_p_^2^ = 0.04). On average, autistic children had lower mean TFF and higher mean blink rate compared to neurotypical children. A main effect of stimulus type was also found for TFF (*F* (1, 440) = 98.17, *P* < 0.0001, η_p_^2^ = 0.18) and blink rate (*F* (1, 440) = 54.30, *P* < 0.0001, η_p_^2^ = 0.12), indicating that, on average, participants exhibited higher TFF and lower blink rate during the social movies compared to nonsocial ones.

**Figure 2:**
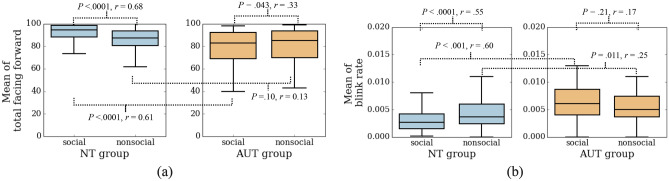
Mean of total facing forward and blink rate for social and nonsocial movies. *NT* neurotypical and *AUT* autistic.

Interaction effects between group and stimulus type were found for both mean TFF (*F* (1, 440) = 28.27, *P* < 0.0001, η_p_^2^ = 0.06) and mean blink rate (*F* (1, 440) = 7.78, *P* = 0.005, η_p_^2^ = 0.02). Comparisons of the mean TFF and blink rate values within the neurotypical and autistic groups during social versus nonsocial movies are shown in Fig. 2. Within-group statistical analysis using Wilcoxon signed-rank test was performed for each of the two groups while comparing the social versus nonsocial movies. The results indicate that the neurotypical children exhibited significantly higher mean TFF (*P* < 0.0001, r = 0.68; Fig. 2a) and lower mean blink rate (*P* < 0.0001, r = 0.55; Fig. 2b), both with large effect sizes, during social movies compared to nonsocial. This potentially indicates higher levels of attentional engagement during the social than the nonsocial movies in the neurotypical group. In contrast, the autistic group had lower mean TFF (*P* = 0.043, r = 0.33; Fig. 2a) during social compared to nonsocial movies with medium effect size and showed no difference in mean blink rate for social versus nonsocial movies (*P* = 0.21, r = 0.17; Fig. 2b).

Examining the differences between the groups using the Mann–Whitney U test for movies of a specific type (social or nonsocial), on average, the neurotypical children exhibited higher mean TFF during the social movies than autistic children (*P* < 0.0001, r = 0.61; Fig. 2), whereas the two groups did not differ in their mean TFF during the nonsocial movies (*P* = 0.1, r = 0.12; Fig. 2) (see also Fig. 1 for line plot of ‘facing forward’ during the task progression). In terms of the mean blink rate, the autistic group exhibited significantly higher mean blink rate than the neurotypical group both during social (*P* < 0.001, r = 0.60; Fig. 2) and nonsocial (*P* = 0.011, r = 0.25; Fig. 2) movies.

To ensure that the overall group difference in TFF was not driving results, we repeated these analyses using only the participants having TFF > 0.80 and found that the pattern of results remained consistent along with statistical significance (see supplementary materials Figs. S1 and S2 for more details and statistics). The number of participants with TFF > 0.80 for the mean TFF and mean blink rate of the social and nonsocial movies are: autistic group (N = 20) and neurotypical group (N = 394). The numbers of participants for each individual movie are presented in Fig. S2 of the supplementary material. Furthermore, to test whether the participant’s age had any effect on the measures, ANCOVA was conducted using ‘age’ as covariate. The pattern of results remained consistent after including the covariate.

**Figure 3:**
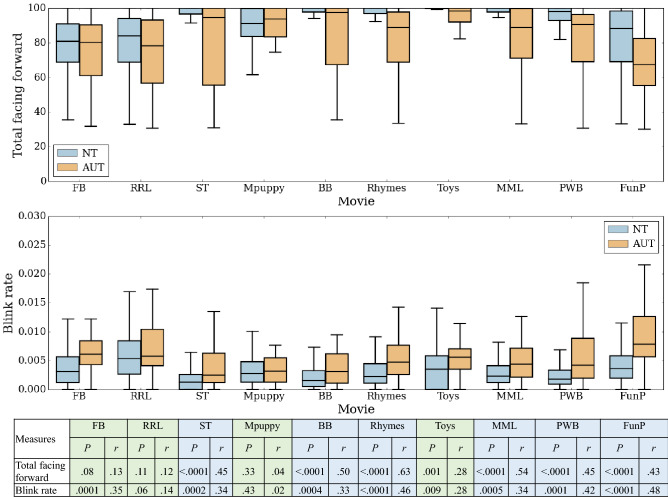
The box plot shows (i) total facing forward and (ii) blink rate for each of the stimuli based on the order in which they were presented. The table shows the respective *P*-values and the effect size (*r*). *NT* neurotypical, *AUT* autistic, *FB* Floating Bubbles, *RRL* Dog in Grass Right-Right-Left, *ST* Spinning Top, *Mpuppy* Mechanical Puppy, *BB* Blowing Bubbles, *MML* Make Me Laugh, *PWB* Playing with Blocks, *FunP* Fun at the Park.

It is possible that the autistic children were facing forward less during the social movies because, on average, the social movies were longer and tended to come toward the end of the app administration, as compared to the nonsocial movies. To address this, group differences in TFF were also examined separately for each individual movie (Fig. 3). For each social movie, even those that were shorter and presented earlier in the sequence rather than toward the end (e.g., “Rhymes”), the difference in TFF between the two groups was significantly different with medium to large effect size (*P*-values and the effect size are presented in Fig. 3), with the autistic group having a reduced TFF. For each nonsocial movie, except for “Toys,” there were no significant differences between the two groups. Thus, even for the nonsocial movie that was of comparable length to the social movies (“Dog in the Grass” = 56 s), the groups did not differ. Additionally, while considering “Toys,” a nonsocial movie, which was presented right after the “Rhymes,” a social movie, the autistic group exhibited a large increase in their ‘facing forward’ (Fig. 1) towards “Toys,” potentially indicating increased attention to dynamic toys, which was not seen for the neurotypical group since they were already ‘facing forward’ during the social movie, “Rhymes”.

Group differences in blink rate were also examined separately for each individual movie (Fig. 3). During each of the social movies, the blink rate was significantly different between the two groups with medium effect size (*P*-values and the effect sizes are presented in Fig. 3); the neurotypical group exhibited lower blink rate than the autistic group during the social movies. For the nonsocial movies, the autistic group showed significantly higher blink rates than the neurotypical for “Floating Bubbles” (medium effect size) and “Toys” (small effect size), but no significant differences were observed during “Dog in Grass Right-Right-Left (RRL)” and “Mechanical Puppy”.

In addition to the estimation of the blink rate (see “Methods and materials”), in the supplementary material we present the (i) valid number of frames (Table S1) and (ii) raw blinks quantity without normalizing with respect to the valid number of frames (Table S2) for both the groups. The blink rate is a normalized representation of the ratio of raw blink quantity and valid number of frames for each participant during a movie since we wanted to have an estimate of blinking only when the participants are ‘facing forward’ towards the movie. However, to ensure that the valid number of frames are not inflating the blink rate, we present a similar statistical analysis for the valid number of frames and the raw blinks quantity (see Tables S1 and S2). The statistically significant differences between the two groups remained the same for the raw blinks quantity. Furthermore, we observed only a moderate correlation (Pearson correlation coefficient, r = − 0.45) between the mean TFF and mean blink rate. This level of correlation indicates that the TFF and blink rate are two different measures that are complementing each other to quantify the participant’s engagement towards the movies.

### Distinguishing groups based on three CVA-based attention measures

We next examined how well the attention measures, mean TFF and mean blink rate, along with mean gaze percent social (MGPS; social attention variable) distinguished the two groups using a classification tool. MGPS was based on the percentage of time the child gazed at the social elements during “Blowing Bubbles” and “Spinning Top” which displayed both social and nonsocial elements separately either on the right or left side of the screen (see “Methods and materials” for details about the movies, and Fig. 1). The MGPS variable was available from a previously published analysis^16^. We have included the MGPS for classification analysis because we excluded the movies “Blowing Bubbles” and “Spinning Top” in the estimation of mean TFF and mean blink rate. Since MGPS gives an estimate of the child’s percentage of look duration towards the social part (left/right) of the screen, we explored its importance in complementing the mean TFF and mean blink rate for classification.

**Figure 4:**
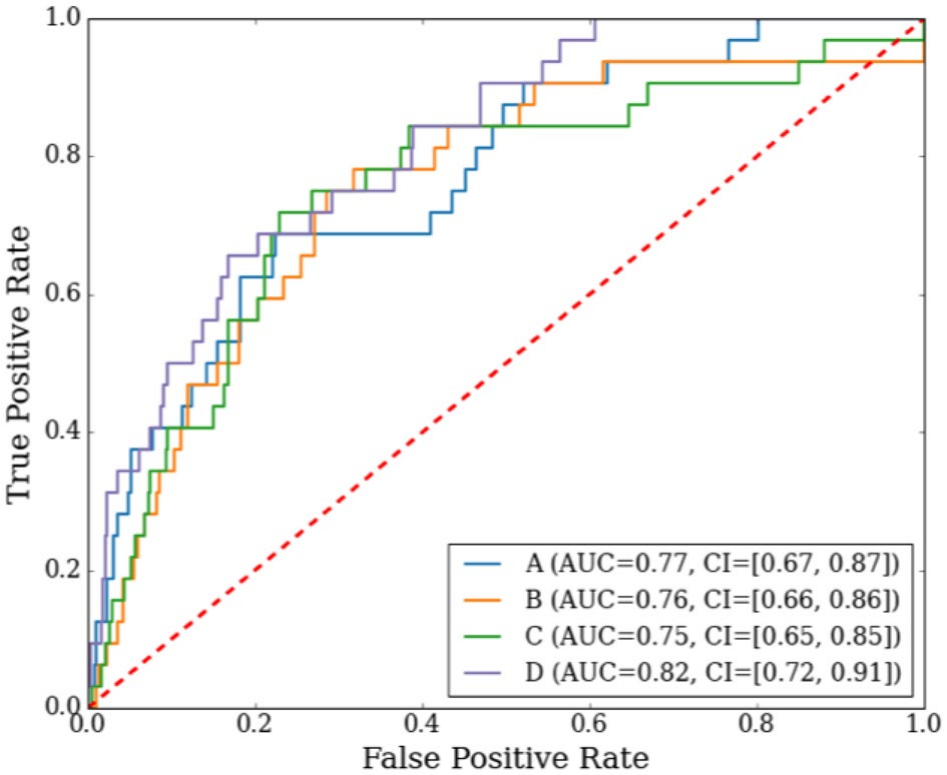
ROC curves using the features individually or in combination. *A* mean TFF_social_, *B* mean blink rate_social_, *C* MGPS, and *D* all the three features.

We considered mean values during social movies (mean TFF_social_ and mean blink rate_social_) for this analysis. These two measures were moderately correlated (negative) with each other (r = − 0.45), when analyzed using the Pearson correlation coefficient. The mean TFF_social_ (r = 0.13) was positively correlated and mean blink rate_social_ (r = − 0.13) was negatively correlated with MGPS. We trained the logistic regression-based classifier using these three attention features and the participant diagnostic group as the classification target to assess how these measures can potentially be used to identify behaviors linked to autism (Fig. 4). Combining the three features achieved a higher area under the curve (AUC) of the receiver operating characteristic (ROC) curve compared to when these features were used individually, indicating that these features complement each other. The confidence intervals of the ROC curves indicate there was an overlap between the individual features and their combination, though the combination still achieved a higher performance.

### Relationship between attention variables and clinical characteristics

For the autistic group, we examined the relationship between the mean TFF and blink rate during the social and nonsocial movies and several clinical variables, including Mullen Early Learning Composite Score and Visual Reception Score, and Autism Diagnostic Observation Schedule (ADOS) Calibrated Severity Scores (ADOS CSS total, restricted/repetitive behavior, social affect). As shown in Table 1, total time facing forward during the social movies was negatively correlated with ADOS total and social affect scores. Autistic children with higher total and social affect ADOS CSS spent less time facing forward during the social movies. Mean total time facing forward (TFF) during the nonsocial, but not the social, movies was negatively correlated with cognitive abilities (Mullen Early Learning Composite Score and Visual Reception Score). Children with higher cognitive abilities spent less time facing forward during the nonsocial movies. We did not find any relationships between the mean blink rate and the clinical variables (Table 1).

**Table 1:** Relationships between attention variables and clinical characteristics for autistic group.

		Clinical measures				
		Mullen scales of early learning		ADOS calibrated severity score		
		Early learning composite score	Visual reception	Restricted repetitive behavior	Social affect	Total
Mean total facing forward	Social	− 0.23	0.08	− 0.16	− 0.57*	− 0.5*
	Nonsocial	− 0.38*	− 0.37*	0.03	− 0.01	− 0.01
Mean blink rate	Social	− 0.14	− 0.09	0.11	0.27	0.26
	Nonsocial	0.1	0.11	0.06	− 0.04	0.01

*P < 0.05, the r values are based on Pearson correlation coefficient.

## Discussion

Research has consistently documented differences in attentional patterns in autistic individuals, characterized by reduced visual social engagement^1^. Such differences are apparent during infancy and offer a means of detecting early signs of autism^14,15,17^. Thus, developing scalable, objective, and quantitative methods for measuring patterns of attentional engagement in infants and toddlers is an important goal.

We have previously shown that CVA can be used to detect distinct patterns of gaze in autistic toddlers, characterized by reduced social attentional engagement, using relatively low-cost, scalable devices without any special set-up, equipment, or calibration^16^. In the present study, we extend this work by demonstrating that using the same app shown on a tablet, we can use CVA to capture distinctive patterns of attentional engagement to social and nonsocial stimuli in autistic toddlers, based on facial orientation and blink rate. This offers an additional quantitative, objective approach to assessing early attention in toddlers.

Overall, autistic toddlers spent less time with their face oriented forward to the movies and exhibited higher blink rates compared to neurotypical toddlers. Our finding of reduced attentional engagement, regardless of stimulus type, is consistent with past work^18^, performed with consumer-grade eye-tracking tools, indicating that reduced visual engagement in autistic toddlers is not limited to social stimuli, but also extends to nonsocial stimuli. This finding is also consistent with eye tracking studies that reported that autistic toddlers exhibit lower overall sustained attention to any dynamic stimuli^19^. A recent review of studies using functional brain imaging to assess social and nonsocial reward processing in autistic individuals suggested that autism is associated with general differences in reward anticipation that are not specific to social stimuli^20^. Considering previous findings linking blink rate to reward circuitry mediated by dopaminergic activity^11,12^, it is possible that differences in blink rate in autistic children found in the present study are associated with alterations in brain circuitry related to reward anticipation while watching the movies.

In addition to overall differences in attentional engagement, autistic and neurotypical toddlers displayed distinctive patterns of attentional engagement when viewing social compared to the nonsocial movies. These results align with previous findings indicating that toddlers later diagnosed with autism tend to exhibit reduced attention to social scenes in free-viewing eye tracking tasks^14^, evident as early as 6 months of age^21^. Neurotypical children faced the screen more often and blinked at a lower rate during social than nonsocial movies, with large effect sizes, suggesting that the social stimuli had higher salience. In contrast, autistic children faced the screen less often during social than nonsocial movies and did not exhibit a differential blink rate to social versus nonsocial movies. This is consistent with a previous study of blink rate which found reduced blink rate in neurotypical children during viewing of social stimuli, possibly due to their increased engagement with the stimuli^13^. Group comparisons showed that, on average, the neurotypical children faced toward the screen more often during the social movies than autistic children, whereas the two groups did not differ in their tendency to face toward the screen during the nonsocial movies. The combination of three different measures of attentional engagement (facing the screen and blink rate during social movies and percent time gazing at social stimuli) distinguished between autistic and neurotypical children with an AUC = 0.82.

Limitations of this study include the sample size, which despite being relatively large, did not offer sufficient power to determine the influence of sex and other demographic characteristics, such as race and ethnicity. Future studies are planned to assess the generalizability of these findings to diverse populations. Such studies are particularly important in light of previous findings linking differences in gaze patterns to face stimuli of same- versus different-race^22,23^. Moreover, future studies will be needed to examine the specificity of the findings to autism by directly comparing blink rate and facial orientation during viewing of social and nonsocial stimuli in autistic children to that of children with other neurodevelopmental disorders, such as ADHD and language or developmental delay.

By combining these novel indices of attention with other digital phenotypic features, such as facial dynamics^24,25^, orienting^26^, and head movements^27,28^, in the future, it may be possible to develop a scalable robust phenotyping tool to detect autism in toddlers, as well as monitor longitudinal development and response to early intervention.

## Methods and materials

### Participants

Participants were 474 toddler age children recruited during their well-child checkup at four pediatric primary care clinics. Based on DSM-5 criteria, 43 toddlers were subsequently diagnosed with autism spectrum disorder. Further, 15 toddlers were diagnosed with language delay/developmental delay, and the remaining 416 participants were neurotypical (NT). Inclusion criteria were: (i) age 16–38 months and (ii) caregiver’s primary language was English or Spanish. Exclusion criteria were: (i) hearing or vision impairments; (ii) the child was too upset or ill during the visit; (iii) the caregiver expressed they had no interest or did not have enough time; (iv) the child would not stay in their caregiver’s lap, or the app or device failed to upload data, or the clinical information was missing; and (v) presence of a significant sensory or motor impairment that precluded the child from watching the movies and/or sitting upright.

#### Ethical considerations

The study protocols were reviewed and approved by the Duke University Health System Institutional Review Board (Pro00085434, Pro00085435). All the methods used in this study were performed in accordance with all relevant guidelines and regulations. Informed consent was obtained from all participants’ parents or their legal guardians. Informed consent was obtained from actors shown in Fig. 1 to publish identifying information/images in an online open-access publication.

### Clinical measures

#### Modified Checklist for Autism in Toddlers: Rrevised with Follow-up (M-CHAT-R/F)

A commonly used screening questionnaire, M-CHAT-R/F^29^ was administered to all the participants. The caregiver-completed M-CHAT-R/F (20 questions) was used to evaluate the presence/absence of autism-related symptoms.

#### Diagnostic and cognitive assessments

Participants whose M-CHAT-R/F score was ≥ 3 initially or had a total score ≥ 2 after the follow-up questions, or whose pediatrician or caregiver expressed developmental concerns, were referred for diagnostic evaluation. The Autism Diagnostic Observation Schedule—Toddler Module (ADOS-2) was administered by a research-reliable licensed psychologist from the study team who determined whether the child met DSM-5 criteria for autism^30^. The Mullen Scales of Early Learning^31^ was used to assess the participant’s cognitive and language abilities.

### Group definitions

#### Autistic (N = 43)

This group included toddlers with an M-CHAT-R/F positive score and/or with developmental concerns raised by the pediatrician/caregiver who subsequently met DSM-5 diagnostic criteria for autism spectrum disorder with or without developmental delay based on both the ADOS-2, Mullen Scales, and clinical judgment by a research reliable psychologist.

#### Neurotypical (N = 416)

This group included toddlers having a high likelihood of typical development with an M-CHAT-R/F score ≤ 1 and no developmental concerns raised by the pediatrician/caregiver, or those who had a positive M-CHAT-R/F score and/or the pediatrician/caregiver raised concerns but then were determined to not have developmental or autism-related concerns by the psychologist based on the ADOS-2, cognitive testing via Mullen Scales, and clinical judgment. Table 2 shows the participants’ demographic characteristics for the autistic and neurotypical groups, consisting of 459 participants.

**Table 2:** Demographic characteristics for neurotypical and autistic groups.

Groups	N (%)	
	Neurotypical (N = 416; 90.63%)	Autistic (N = 43; 9.37%)
Age in months		
Mean (SD)	20.59 (3.18)^a^	24.32 (4.64)^a^
Sex		
Boy	209 (50.24%)^b^	32 (74.42%)^b^
Girl	207 (49.76%)^b^	11 (25.58%)^b^
Race		
American Indian/Alaskan Native	1 (0.24%)	3 (6.97%)
Asian	6 (1.44%)	1 (2.32%)
Black or African American	43 (10.33%)	6 (13.95%)
Native Hawaiian or Other Pacific Islander	0 (0.00%)	0 (0.00%)
White/Caucasian	316 (75.96%)	22 (51.16%)
More than one race	41 (9.85%)	7 (16.28%)
Other	9 (2.16%)	4 (9.30%)
Ethnicity		
Hispanic/Latino	31 (7.45%)^b^	13 (30.23%)^b^
Not Hispanic/Latino	385 (92.54%)^b^	30 (69.77%)^b^
Caregiver's highest level of education		
Without high school diploma	2 (0.49%)^b^	4 (9.30%)^b^
High school diploma or equivalent	14 (3.36%)^b^	6 (13.95%)^b^
Some college education	40 (9.61%)^b^	10 (23.25%)^b^
4-year college degree or more	356 (85.57%)^b^	23 (53.48%)^b^
Unknown/not reported	4 (0.96%)	0 (0.00%)

Clinical variables	Mean (SD)	
ADOS-2 Toddler Module		
Calibrated Severity Score	–	7.60 (1.67)
Mullen Scales of Early Learning		
Early Learning Composite Score	–	63.15 (9.94)
Expressive Language T-score	–	28.02 (7.25)
Receptive Language T-score	–	22.90 (4.81)
Fine Motor T-score	–	33.97 (10.40)
Visual Reception T-score	–	33.22 (10.67)

The age (in months) at which participants received their diagnosis (ADOS-2): M = 23.9, SD = 4.5.

The interval (in months) between the age at diagnosis and the app administration: M = 0.7, SD = 1.2.

*ADOS-2* Autism Diagnostic Observation Schedule—Second Edition.

^a^Significant difference between the two groups based on ANOVA test.

^b^Significant difference between the two groups based on Chi-Square test.

There was another group of participants (N = 15) who had a positive M-CHAT-R/F score and received a diagnosis of language delay/developmental delay (LD-DD) without autism. Children included in the LD-DD group were those who had failed the M-CHAT-R/F or had provider or caregiver developmental concerns, were referred for evaluation and administered the ADOS-2 and Mullen Scales and were then determined by a licensed psychologist not to meet DSM-5 criteria for autism. All children in the LD-DD group scored ≥ 9 points below the mean on at least one Mullen Early Learning Subscale (1 SD = 10 points). Given the small sample size, we present data for the LD-DD group only in the supplementary materials (refer to Table S1, Figs. S3 and S4). The demographic characteristics of 474 participants, including the LD-DD participants, are presented in Table S3.

### Application (app) administration and stimuli

The app was administered on a tablet (iPad) that displayed developmentally appropriate, short social and nonsocial movies during the child’s well-child visit. The tablet was mounted on a tripod placed at ~ 60 cm from the child while the caregiver was holding the child on their lap. Any other family members (e.g., siblings) and the research staff who administered the app stayed behind both the caregiver and the child. The tablet’s frontal camera recorded the video of the child at 30 fps which was further used for CVA to automatically capture their behavioral responses. The social and nonsocial movies were presented in the same order for all participants, as described next. The total duration of the movies was about 8 min. All movies contained both visual and auditory stimuli, described below. In both the social and nonsocial movies, visual and auditory stimuli were sometimes synchronized (e.g., "Dog in the Grass" and "Rhymes") and sometimes non-synchronized (e.g., "Floating Bubbles and "Make Me Laugh"). Nonsocial movies contained dynamic objects with sound, unlike the social movies that had higher social content with ethnically and racially diverse human actors in the scenes. All the social movies depicted human actors. The language used by the actors was provided in English or Spanish depending on the child’s primary language at home. Figure 1 shows a snapshot of the movies.

1. *Floating Bubbles (35 s; nonsocial)*. Bubbles move randomly throughout the frame of the screen with a gurgling sound.
2. *Dog in Grass (16 s; nonsocial)*. In the first part of this movie, a cartoon barking puppy appears at the center and the four corners of the screen.
3. *Dog in Grass Right-Right-Left (RRL) (40 s; nonsocial)*. In the second part of this movie, the barking puppy appears randomly in the right/left side of the screen at first, followed by a constant right-right-left (RRL) pattern. Total length of Dog in Grass = 56 s.
4. *Spinning Top (53 s; social)*. An actress plays with a spinning top with successful and unsuccessful attempts at spinning, looks towards the screen to convey eye contact, smiles, frowns, and makes a few verbal expressions in English or Spanish.
5. *Mechanical Puppy (25 s; nonsocial)*. A mechanical toy puppy barks, jumps, and walks towards a group of toys.
6. *Blowing Bubbles (64 s; social)*. An actor with a bubble wand blows bubbles with successful and unsuccessful attempts blowing, along with smiling and frowning, and looks towards the screen to convey eye contact with a few verbal expressions in English or Spanish.
7. *Rhymes (30 s; social)*. An actress says nursery rhymes such as Itsy-Bitsy Spider in English or Spanish with smiles and gestures.
8. *Toys (19 s; nonsocial)*. Dynamic toys with sound are shown.
9. *Make Me Laugh (56 s; social)*. An actress demonstrates silly, funny actions with smiling and eye contact.
10. *Playing with Blocks (71 s; social)*. Two child actors, a boy and a girl, interact and play with toys with occasional verbalizations in English or Spanish.
11. *Fun at the Park (51 s; social)*. Two actresses stand at each side of the frame, having a turn-taking conversation in English or Spanish with no gestures.

### Estimation of ‘facing forward’ and blink rate variables

We first used CVA to determine the amount of time the child’s face was oriented toward the screen of the device (‘facing forward’). A face detection algorithm^32^ was used to capture the child’s face in each frame of the recorded video. In order to track only the participant’s face and ignore all other faces in the frame, we performed a semi-supervised face detection algorithm (for details, see Refs.^16,26^). Subsequently, we extracted 49 facial landmark points consisting of 2D-positional coordinates^33^ that were time-synchronized with the movies. Using the facial landmarks, for each frame, we computed the child’s head pose angles relative to the tablet’s frontal camera such as *θ*_*yaw*_ (left–right), *θ*_*pitch*_ (up-down), and *θ*_*roll*_ (tilting left–right) (as described in Ref.^34^).

#### Facing forward

A child’s orientation towards the screen, i.e. ‘facing forward’ during any given frame was defined using their (i) head pose angle, (ii) eye gaze, and (iii) rapidity in head movement. The child’s head pose |*θ*_*yaw*_|< 25° was used, acting as a proxy for attentional focus on the screen, consistent with our previous work^27,34^, which is supported by the central bias theory for gaze estimation^35,36^. Then, for each frame, we checked if the estimated gaze of the participant was on the tablet’s screen and if their eyes were open. The participant’s gaze information was extracted using an automatic gaze estimation algorithm based on a pre-trained deep neural network^16,37^. Finally, we excluded the frames where the head was moving rapidly (this can lead to errors in the CVA). To this end, we first performed smoothing of the head pose signal *θ*_*yaw*_, obtaining *θ*_*yaw*_*’*. The head was considered to be moving rapidly if at any point *θ*_*yaw*_*’* of the current frame was > 150% of the previous frame. Finally, the total facing forward variable (TFF) was estimated as a percentage of frames ‘facing forward’ out of the number of frames for each movie (ranging between 0 and 100). Details on the algorithm are presented in the supplementary materials, Algorithm S1.

#### Blink rate

We estimated the participant’s number of blinks while they were watching each of the presented movies, as described next. OpenFace, a facial analysis toolkit^38^ that offers facial action units on a frame-by-frame basis, was used. These action units are based on the standard facial action coding system^39^. For the blinking action, we used action unit 45 (AU45) to estimate the participant’s blinks. A smoothing of the AU45 time-series signal was performed, followed by detecting the number of peaks, which are associated with blink actions (see supplementary materials, Algorithm S2). To obtain the blink rate (blink rate), we normalized the number of blinks with respect to the number of valid frames. The valid frames were defined as frames during which the participant was (i) ‘facing forward’ (see above) and (ii) the confidence outcome of the OpenFace was at or above the recommended threshold (i.e. 0.75)^38^.

### Social attention variable using eye gaze estimation

The “Spinning Top” and “Blowing Bubbles” stimuli had equally spatially halved representations of social (actor/actress) and nonsocial (toys/bubbles) components on the right or left side of the screen (see Fig. 1). For these two movies, we computed the percentage of the time the participants gazed toward the social/nonsocial portion of the screen. The average gaze towards the social portion across the two movies was referred to as mean gaze percent social (MGPS). Previous work by our team based on this app^16^ showed that autistic toddlers looked significantly less to the side of the screen that displayed the social elements compared to neurotypical toddlers.

### Statistical analysis

A 2X2 mixed ANOVA was used to estimate the main effects due to (i) participant group and (ii) movie type (social and nonsocial) and their interaction effects via the Python method *pinguouin.mixed_anova* from Pingouin package version 0.5.2^40^*.* The Mann–Whitney U test was used to estimate the statistical significance between the groups, using Python method *pingouin.mwu*. Withingroup comparisons were performed using the Wilcoxon signed-rank test using *pingouin.wilcoxon*. The statistical power was presented with effect size, ‘r’ for *pingouin.mwu* and *pingouin.wilcoxon*, and ‘η_p_^2^’ for ANOVA. Additionally, analysis of covariance (ANCOVA) using *pingouin.ancova* was performed to determine the influence of covariates. To assess the contribution of the three attention features (TFF, blink rate, and MGPS) either individually or in combination to distinguish the autistic and neurotypical groups, we used a linear logistic regression from sklearn Python package version 0.23.2^41^. The classification performance was compared using the area under the curve of the receiver operating characteristic considering leave-one-out cross-validation^42^. Using the Hanley and McNeil method^43^, we have presented the 95% confidence interval (CI).

## Supplementary Information

Supplementary Information.

## Data Availability

Data that support the findings of this study are available from the corresponding authors upon request and following IRB and privacy regulations.

## References

[1] Klin A, Shultz S, Jones W (2015). Social visual engagement in infants and toddlers with autism: Early developmental transitions and a model of pathogenesis. Neurosci. Biobehav. Rev..

[2] Chita-Tegmark M (2016). Social attention in ASD: A review and meta-analysis of eye-tracking studies. Res. Dev. Disabil..

[3] Setien-Ramos I (2022). Eye-tracking studies in adults with autism spectrum disorder: A systematic review and meta-analysis. J. Autism Dev. Disord..

[4] Ortega J, Plaska CR, Gomes BA, Ellmore TM (2022). Spontaneous eye blink rate during the working memory delay period predicts task accuracy. Front. Psychol..

[5] Oh J, Jeong SY, Jeong J (2012). The timing and temporal patterns of eye blinking are dynamically modulated by attention. Hum. Mov. Sci..

[6] Rac-Lubashevsky R, Slagter HA, Kessler Y (2017). Tracking real-time changes in working memory updating and gating with the event-based eye-blink rate. Sci. Rep..

[7] Ranti C, Jones W, Klin A, Shultz S (2020). Blink rate patterns provide a reliable measure of individual engagement with scene content. Sci. Rep..

[8] Hoppe D, Helfmann S, Rothkopf CA (2018). Humans quickly learn to blink strategically in response to environmental task demands. Proc. Natl. Acad. Sci. U. S. A..

[9] Groen Y, Börger NA, Koerts J, Thome J, Tucha O (2017). Blink rate and blink timing in children with ADHD and the influence of stimulant medication. J. Neural Transm..

[10] Reddy VC, Patel SV, Hodge DO, Leavitt JA (2013). Corneal sensitivity, blink rate, and corneal nerve density in progressive supranuclear palsy and Parkinson disease. Cornea.

[11] Roberts JE, Symons FJ, Johnson AM, Hatton DD, Boccia ML (2005). Blink rate in boys with fragile X syndrome: Preliminary evidence for altered dopamine function. J. Intellect. Disabil. Res..

[12] Hornung T, Chan WH, Müller RA, Townsend J, Keehn B (2019). Dopaminergic hypo-activity and reduced theta-band power in autism spectrum disorder: A resting-state EEG study. Int. J. Psychophysiol..

[13] Shultz S, Klin A, Jones W (2011). Inhibition of eye blinking reveals subjective perceptions of stimulus salience. Proc. Natl. Acad. Sci. U. S. A..

[14] Chawarska K, Macari S, Shic F (2013). Decreased spontaneous attention to social scenes in 6-month-old infants later diagnosed with autism spectrum disorders. Biol. Psychiatry.

[15] Jones W, Klin A (2013). Attention to eyes is present but in decline in 2-6-month-old infants later diagnosed with autism. Nature.

[16] Chang Z (2021). Computational methods to measure patterns of gaze in toddlers with autism spectrum disorder. JAMA Pediatr..

[17] Jones EJH (2016). Reduced engagement with social stimuli in 6-month-old infants with later autism spectrum disorder: A longitudinal prospective study of infants at high familial risk. J. Neurodev. Disord..

[18] McLaughlin CS (2021). Reduced engagement of visual attention in children with autism spectrum disorder. Autism.

[19] Chawarska K, Ye S, Shic F, Chen L (2016). Multilevel differences in spontaneous social attention in toddlers with autism spectrum disorder. Child Dev..

[20] Keifer CM, Day TC, Hauschild KM, Lerner MD (2021). Social and nonsocial reward anticipation in typical development and autism spectrum disorders: Current status and future directions. Curr. Psychiatry Rep..

[21] Shic F, Macari S, Chawarska K (2014). Speech disturbs face scanning in 6-month-old infants who develop autism spectrum disorder. Biol. Psychiatry.

[22] Krasotkina A, Götz A, Höhle B, Schwarzer G (2020). Infants’ gaze patterns for same-race and other-race faces, and the other-race effect. Brain Sci..

[23] Pickron CB, Fava E, Scott LS (2017). Follow my gaze: Face race and sex influence gaze-cued attention in infancy. Infancy.

[24] Carpenter KLH (2021). Digital behavioral phenotyping detects atypical pattern of facial expression in toddlers with autism. Autism Res..

[25] Krishnappa Babu PR (2021). Exploring complexity of facial dynamics in autism spectrum disorder. IEEE Trans. Affect. Comput..

[26] Perochon S (2021). A scalable computational approach to assessing response to name in toddlers with autism. J. Child Psychol. Psychiatry Allied Discip..

[27] Dawson G (2018). Atypical postural control can be detected via computer vision analysis in toddlers with autism spectrum disorder. Sci. Rep..

[28] Krishnappa Babu PR (2023). Complexity analysis of head movements in autistic toddlers. J. Child Psychol. Psychiatry.

[29] Robins DL (2014). Validation of the modified checklist for autism in toddlers, revised with follow-up (M-CHAT-R/F). Pediatrics.

[30] Luyster R (2009). The autism diagnostic observation schedule - Toddler module: A new module of a standardized diagnostic measure for autism spectrum disorders. J. Autism Dev. Disord..

[31] Mullen EM (1995). Mullen scales of early learning. Circ. Pines MN Am. Guid. Serv..

[32] King DE (2009). Dlib-ml: A machine learning toolkit. J. Mach. Learn. Res..

[33] De La Torre, F. *et al.* IntraFace. in *2015 11th IEEE International Conference and Workshops on Automatic Face and Gesture Recognition, FG 2015* 1–8 (2015). doi:10.1109/FG.2015.7163082. 10.1109/FG.2015.7163106 PMC4893200 27275131

[34] Hashemi J (2021). Computer vision analysis for quantification of autism risk behaviors. IEEE Trans. Affect. Comput..

[35] Li, Y., Fathi, A. & Rehg, J. M. Learning to predict gaze in egocentric video. in *Proceedings of the IEEE International Conference on Computer Vision* 3216–3223 (2013). doi:10.1109/ICCV.2013.399.

[36] Mannan S, Ruddock KH, Wooding DS (1995). Automatic control of saccadic eye movements made in visual inspection of briefly presented 2-D images. Spat. Vis..

[37] Krafka K, Krafka K (2016). Eye tracking for everyone. Proceedings of the IEEE Computer Society Conference on Computer Vision and Pattern Recognition.

[38] Baltrusaitis, T., Zadeh, A., Lim, Y. C. & Morency, L. P. OpenFace 2.0: Facial behavior analysis toolkit. in *Proceedings - 13th IEEE International Conference on Automatic Face and Gesture Recognition, FG 2018* 59–66 (2018). doi:10.1109/FG.2018.00019.

[39] Ekman, P. & Wallace, V. F. Facial Action Coding System (FACS). APA PsycTests. 10.1037/t27734-000 (1978).

[40] Vallat R (2018). Pingouin: Statistics in Python. J. Open Source Softw..

[41] Pedregosa F (2011). Scikit-learn: Machine learning in Python. J. Mach. Learn. Res..

[42] Elisseeff A, Pontil M (2003). Leave-one-out error and stability of learning algorithms with applications. NATO Sci. Ser. III Comput. Syst. Sci..

[43] Hanley JA, McNeil BJ (1982). The meaning and use of the area under a receiver operating characteristic (ROC) curve. Radiology.

