# Supplementary material for: Blink rate and facial orientation reveal distinctive patterns of attentional engagement in autistic toddlers: a digital phenotyping approach
Source: Sci Rep. 2023 May 3;13:7158. doi: 10.1038/s41598-023-34293-7 (PMC10156751; doi:10.1038/s41598-023-34293-7)
Supplement: Supplementary file 1 — Supplementary Information. [file 41598_2023_34293_MOESM1_ESM.docx]

**Supplementary Material**

**Blink rate and facial orientation reveal distinctive patterns of attentional engagement in autistic toddlers: A digital phenotyping approach**

Pradeep Raj Krishnappa Babu^1, #^, Vikram Aikat^2,#^, J. Matias Di Martino^1^, Zhuoqing Chang^1^, Sam Perochon^3^, Steven Espinosa^4^, Rachel Aiello^5,6^, Kimberly L.H. Carpenter^5,6^, Scott Compton^5,6^, Naomi Davis^5,6^, Brian Eichner^7^, Jacqueline Flowers^5,6^, Lauren Franz^5,6,8^, Geraldine Dawson^5,6,*^, and Guillermo Sapiro^1,9,*^

^1^Department of Electrical and Computer Engineering, Duke University, Durham, NC, USA

^2^Department of Computer Science, Duke University, Durham, NC, USA

^3^Ecole Normale Supérieure Paris-Saclay, Gif-Sur-Yvette, France

^4^Office of Information Technology, Duke University, Durham, NC, USA

^5^Department of Psychiatry and Behavioral Sciences, Duke University, Durham, NC, USA

^6^Duke Center for Autism and Brain Development, Duke University, Durham, NC, USA

^7^Department of Pediatrics, Duke University, Durham, NC, USA

^8^Duke Global Health Institute, Duke University, Durham, NC, USA

^9^Departments of Biomedical Engineering, Mathematics, and Computer Science, Duke University, Durham, NC, USA

^#^First authors

*Co-Senior authors

E-mail: [geraldine.dawson@duke.edu](mailto:geraldine.dawson@duke.edu); [guillermo.sapiro@duke.edu](mailto:guillermo.sapiro@duke.edu)

**Analysis of participants who ‘faced forward’ for 80% of the movie duration**

An analysis of blink rate was conducted to determine whether similar results are obtained when we only include participants who faced the screen (total facing forward; TFF) 80% of the time. As shown in Figure S1 and Figure S2, results were consistent with those in Figures 2 and 3, respectively. The statistically significant differences between and within the groups are presented in Figure S1 and within the table of Figure S2.

(a) (b)

**Figure S1.** Mean of total facing forward and blink rate for social and non-social movies for participants whose total facing forward was > 80%. Note: NT = neurotypical and AUT = autistic.

**Figure S2.** The box plot shows the measures (i) total facing forward and (ii) blink rate for each of the stimuli based on the order in which they were presented. The table shows the respective *P*-values and the effect size (*r*); the last row of the table represents the sample size in the two groups whose total facing forward was >80% for each specific movie. Note: NT = neurotypical, AUT = autistic, FB = Floating Bubbles, RRL = Dog in Grass Right-Right-Left, ST = Spinning Top, Mpuppy = Mechanical Puppy, BB = Blowing Bubbles, MML = Make Me Laugh, PWB = Playing with Blocks, and FunP = Fun at the Park.

**Statistics on valid number of frames and raw blink quantity**

| *Table S1: Percentage of valid frames used for blink rate computation* | | |
| --- | --- | --- |
| Movies | Autistic | Neurotypical |
|  | Mean (SD)% | Mean (SD)% |
| Social  Spinning Top**  Blowing Bubbles**  Rhymes**  Make Me Laugh**  Playing with Blocks**  Fun at the Park**  Nonsocial  Floating Bubbles**  Dog in Grass RRL*  Mechanical Puppy  Toys** | 77.2 (24.4)%  81.3 (24.4)%  45.8 (14.8)%  79.8 (22.8)%  73.6 (27.8)%  62.1 (19.7)%  68.2 (21.1)%  68.1 (25.2)%  81.3 (24.4)%  32.5 (6.1)% | 95.3 (10.5)%  95.6 (10.9)%  58.1 (5.7)%  95.6 (10.9)%  92.1 (12.5)%  77.6 (21.7)%  71.7 (19.9)%  76.6 (19.4)%  83.1 (16.1)%  36.2 (4.7)% |
| ** = *P* <0.0001 and * = *P*<0.5 | | |

| *Table S2: Number of raw blinks quantity without normalization with respect to valid frames* | | |
| --- | --- | --- |
| Movies | Autistic | Neurotypical |
|  | Mean (SD)% | Mean (SD)% |
| Social  Spinning Top**  Blowing Bubbles**  Rhymes**  Make Me Laugh**  Playing with Blocks**  Fun at the Park**  Nonsocial  Floating Bubbles**  Dog in Grass RRL  Mechanical Puppy  Toys* | 4.9 (5.3)%  5.8 (4.5)%  3.8 (2.5)%  6.6 (5.3)%  6.5 (4.4)%  7.9 (5.5)%  4.5 (3.6)%  5.7 (3.8)%  2.2 (2.2)%  2.6 (1.6)% | 3.2 (4.5)%  4.5 (5.5)%  2.6 (3.0)%  5.1 (5.2)%  4.9 (4.9)%  4.8 (3.7)%  2.7 (2.5)%  5.6 (4.4)%  2.2 (2.18)%  2.3 (2.3)% |
| ** = *P* <0.0001 and * = *P*<0.5 | | |

Table S1 presents the mean and standard deviation of the percentage of valid frames used for the blink rate computation for each of the groups. Similarly, Table S2 indicates the raw blinks quantitites for both the groups. When comparing the two groups for each different movie using a statistical test (Mann–Whitney U test), both the (i) percentage of valid frames and (ii) raw blink quantity had similar statistical significance as blink rate.

**Results for children with language delay/development delay (LD-DD)**

Table S3 shows the details of all the participants (neurotypical, autistic, and LD-DD). Figure S3 shows the mean total facing forward (TFF) and mean blink rate for the social and nonsocial stimuli. The distribution of the LD-DD group appears to have similar attentional patterns as that of neurotypical group, unlike the autistic group, indicating the potential specificity of the proposed CVA-based measures for autism. Results for the TFF and blink rate for individual movies are presented in Figure S4. The statistical results for the individual movies in Figure S4 (*P*-value and effect sizes) are presented by comparing the autistic and LD-DD groups only. Overall, the distribution of the LD-DD group was observed to be different from the autistic and similar to the neurotypical group.

(a)

(b)

**Figure S3.** Mean of total facing forward and blink rate for social and non-social movies. NT = neurotypical and AUT = autistic.

**Figure S4.** The box plot shows the measures (i) total facing forward and (ii) blink rate for each of the stimuli based on the order in which they were presented. The table shows the respective *P*-values and the effect size (*r*), while comparing the autistic and LD-DD groups. Note: NT = Neurotypical, AUT = Autism, FB = Floating Bubbles, RRL = Dog in Grass Right-Right-Left, ST = Spinning Top, Mpuppy = Mechanical Puppy, BB = Blowing Bubbles, MML = Make Me Laugh, PWB = Playing with Blocks, FunP = Fun at the Park

| Table S3: *Participant demographic characteristics* | | | | | |  |
| --- | --- | --- | --- | --- | --- | --- |
|  | **N (%)** | | | | |  |
| Groups | Neurotypical  (N=416; 87.76%) | Autistic  (N=43; 9.07%) | | | LD-DD  (N=15; 3.17%) | |
| Age in months  Mean (SD) | 20.59 (3.18)^a^ | 24.32 (4.64)^a^ | | | 22.62 (3.48) | |
| Sex  Male  Female | 209 (50.24%)^b^  207 (49.76%)^b^ | 32 (74.42%)^b^  11 (25.58%)^b^ | | | 12 (74.42%)^b^  3 (25.58%)^b^ | |
| Race  American Indian/Alaskan Native  Asian  Black or African American  Native Hawaiian or Other Pacific  Islander  White/Caucasian  More Than One Race  Other  Unknown/declined | 1 (0.24%)  6 (1.44%)  43 (10.33%)  0 (0.00%)  316 (75.96%)  41 (9.85%)  9 (2.16%)  0 (0.00%) | 3 (6.97%)  1 (2.32%)  6 (13.95%)  0 (0.00%)  22 (51.16%)  7 (16.28%)  4 (9.30%)  0 (0.00) | | | 0 (0.00%)  0 (0.00%)  5 (33.33%)  0 (0.00%)  7 (46.67%)  0 (0.00%)  2 (13.33%)  1 (6.67) | |
| Ethnicity  Hispanic/Latino  Not Hispanic/Latino | 31 (7.45%)^b^  385 (92.54%)^b^ | 13 (30.23%)^b^  30 (69.77%)^b^ | | | 5 (33.33%)^b^  10 (66.67%)^b^ | |
| Caregivers’ Highest Level of Education  Without High School Diploma  High School Diploma or Equivalent  Some College Education  4-Year College Degree or More  Unknown/Not Reported | 2 (0.49%)^b^  14 (3.36%)^b^  40 (9.61%)^b^  356 (85.57%)^b^  4 (0.96%) | 4 (9.30%)^b^  6 (13.95%)^b^  10 (23.25%)^b^  23 (53.48%)^b^  0 (0.00%) | | | 4 (26.67%)^b^  5 (33.33%)^b^  0 (0.00%)^b^  6 (40.00%)^b^  0 (0.00%) | |
| Clinical Variables | **Mean (SD)** | | | | |  |
| ADOS-2 Toddler Module  Calibrated Severity Score | - | | 7.60 (1.67) | 4.06 (1.38) | | |
| Mullen Scales of Early Learning  Early Learning Composite Score  Expressive Language T-Score  Receptive Language T-Score  Fine Motor T-Score  Visual Reception T-Score | -  -  -  -  - | | 63.15 (9.94)  28.02 (7.25)  22.90 (4.81)  33.97 (10.40)  33.22 (10.67) | 72.53 (14.93)  35.06 (10.28)  31.13 (12.41)  38.46 (6.18)  35.80 (11.79) | | |
| ADOS-2: Autism Diagnostic Observation Schedule – Second Edition  ^a^ Significant difference between the two groups based on ANOVA test.  ^b^ Significant difference between the two groups based on Chi-Square test. | | | | | | |

**Pseudocode for estimating the features via computer vision analysis**

| **Algorithm S1: Facing forward** |
| --- |
| *CalculateFacingForward (partcipant’s video, θ_yaw_, gaze):*  *for a_frame in participant’s video:*  *if “\| θ_yaw_ \| < 25°” and*  *“gaze lied within the screen” and*  *“θ_yaw_’ in the current frame <150% of θ_yaw_’ in the previous frame”:*  *then facing_forward[a_frame] = 1*  *else:*  *facing_forward[a_frame] = 0*  *return the array of facing_forward*  *CalculateTotalFacingForward (facing_forward):*  *(sum of the 1’s in the facing_forward / duration of the movie stimuli in number of frames) * 100* |

| **Algorithm S2: Blink rate** |
| --- |
| *CalculateBlinkRate (AU45 time series, OpenFace confidence value, facing_forward):*  *# Filter Step*  *valid_frames = 0*  *for a_frame in video:*  *if “facing_forward[a_frame] == 1” and*  *“OpenFace confidence value < .75”:*  *then valid_frames += 1*  *else:*  *do nothing*  *# Peak Detection Step*  *run python scipy peak detection on filtered AU45 time series:*  *return number of peaks*  *blink_rate = number of peaks / valid_frames*  *return blink rate* |
